# Editorial: Horizontal gene transfer mediated bacterial antibiotic resistance, volume II

**DOI:** 10.3389/fmicb.2023.1221606

**Published:** 2023-06-23

**Authors:** Dongchang Sun, Xingmin Sun, Yongfei Hu, Yoshiharu Yamaichi

**Affiliations:** ^1^College of Biotechnology and Bioengineering, Zhejiang University of Technology, Hangzhou, China; ^2^Department of Molecular Medicine, Morsani College of Medicine, University of South Florida, Tampa, FL, United States; ^3^State Key Laboratory of Animal Nutrition, College of Animal Science and Technology, China Agricultural University, Beijing, China; ^4^Université Paris-Saclay, CEA, CNRS, Institute for Integrative Biology of the Cell (I2BC), Gif-sur-Yvette, France

**Keywords:** horizontal gene transfer (HGT), bacterial antibiotic resistance, antibiotic resistance genes (ARGs), plasmid, bacterial conjugation, mobile genetic elements (MGEs)

The issue of bacterial antibiotic resistance (BAR) has reached an alarming level in recent years. In 2019 alone, BAR was responsible for the deaths of approximately five million people globally, making it the third leading cause of death (Antimicrobial Resistance Collaborators, 2022). This figure surpasses the previous estimation (WHO, 2014), highlighting the severity of the situation. Bacteria can easily transfer antibiotic resistance genes (ARGs) through horizontal gene transfer (HGT), which can promote the development and spread of resistance within the bacterial population (Sun et al., 2019). Ten publications have recently been published on the topic of “Horizontal Gene Transfer Mediated Bacterial Antibiotic Resistance II,” presenting the latest findings and advancements in this field. These publications could serve as new reference points to tackle BAR in the future.

In this Research Topic, three reports (Xin et al.; Zhang S. et al.; Yang et al.) demonstrated the high prevalence of ARGs in different regions of China, revealing the serious challenge of BAR in the country. By evaluating 4, 414 strains of enterococci from hospitals in 26 provinces in China, Xin et al. demonstrated the wide distribution of the fosfomycin resistance gene *fosX* in *E. faecium*, a human pathogen responsible for gastrointestinal tract infections. ARGs *tet(X), bla*, and *mcr* threaten the efficacy of tigecycline, carbapenems, and colistin, which represent the three last-resort antibiotics. Zhang S. et al. summarized the distribution of *tet(X)* genes in China and demonstrated the presence of *tet(X)* genes in 24 provinces. Remarkably, *tet(X)* genes were identified not only in humans, livestocks, poultries and aquatic animals but also in wild animals and the environment, reflecting the fast spread of this ARG in the wild in China. Yang et al. reported not only the presence of *bla*_NDM − 5_ and *mcr-8.1* in an animal breeding area in eastern China but also the clonal transfer of the two ARGs and their potential dissemination through horizontal gene transfer. These studies highlight the urgency of taking action to combat BAR in China.

Although observational studies have manifested the present situation of BAR, our understanding of this issue largely relies on retrospective analysis. A review article in this Research Topic summarized the recent progress of *in silico, in vitro*, and *in vivo* modeling of HGT, providing new perspectives and directions on understanding the plasmid-mediated transfer of ARGs in the gut of animals (Ott and Mellata). In nature, ARGs are often disseminated by vehicles (e.g., plasmid) and mobile genetic elements (MGEs). Guzman-Otazo et al. found that IncN plasmids can transmit ARGs via conjugation from environmental waterborne bacteria to *Escherichia coli*, which colonizes in the intestine of terrestrial animals, enabling the spread of ARGs across aquatic and terrestrial environments. Integrative and conjugative elements (ICEs) and integrons are MGEs that mediate the dissemination of ARGs. Two studies in this Research Topic investigated the roles of ICEs and integrons in facilitating the spread of ARGs. Through whole-genome analysis of 27 multidrug-resistant (MDR) *Proteus* strains, Li Y. et al. revealed that nine ICEs shared a common backbone structure, implicating the dissemination of ICEs in *Proteus* strains from livestocks and poultries and humans. Their study also revealed that ARGs were carried by MGEs of genetic diversity, highlighting the necessity of continuous monitoring. Bitar et al. analyzed 32 VIM metallo-β-lactamase producing Enterobacterales from Czech hospitals, and sequenced 19 isolates. Their work revealed that the spread of VIM-encoding integron In110 was more prevalent than other VIM-encoding integrons and that many new ARGs-carrying MGEs could be evolving.

Compared with that in non-pathogenic bacteria, the transfer of ARGs in pathogens is more threatening. Two studies in this Research Topic showed that the impact of HGT on the evolution of pathogens could be species-specific. Li P. et al. reported that outer membrane vesicles were able to incorporate DNA and deliver both virulence and antimicrobial-resistant plasmids. Their work provides new evidence supporting that HGT plays an important role in facilitating the evolution of carbapenem-resistant hypervirulent *Klebsiella pneumonia*. Whereas, Kandinov et al. discovered that the plasmid expressing *bla*_TEM − 20_ reduced the viability of *Neisseria gonorrhoeae*. This could result from the expression of extended-spectrum β-lactamase, which may affect the structure of the peptidoglycan layer, providing an explanation for the absence of clinical isolates of extensive spectrum beta-lactamase-producing *N. gonorrhoeae*. Investigation of the transfer of ARGs in pathogens would not only explain co-evolutionary relationships between resistance and virulence but also provide guidance for the clinical use of antibiotics.

To be stably inherited, horizontally transferred ARGs need to be maintained in either a plasmid or the genome of the recipient bacteria through genetic recombination. Zhang G. et al. characterized an integrase of genomic island GI*sul2*, which mediated the integration of MGEs through site-specific recombination in *Shigella flexneri*. They found that GI*sul2* can excise and integrate GI*sul2* and the IS*CR*-related element CR2-*sul2* unit by site-specific recombination between the host chromosomal attachment sites, suggesting a potential role of GI*sul2* integrase homologs in the dissemination of GI*sul2* units.

In summary, articles of this Research Topic demonstrated the prevalence of ARGs, diverse mechanisms of transfer of ARGs under different settings, and novel ways of integration of MGEs carrying ARGs. Although remarkable advances in the knowledge of the dissemination of ARGs have been made in recent years, we still know little about associations between environmental cues and the HGT-driven spread of ARGs under different environmental conditions. More importantly, despite the severity of BAR being more serious than estimated, there is a lack of effective strategies to tackle this problem. To address this global crisis, collaboration among different fields from countries all over the world remains in high demand.

We would like to acknowledge all participating authors for their contributions, reviewers for their constructive comments, and our editorial team members for their great efforts.

## Author contributions

All authors participated in editing manuscripts in this Research Topic, reviewed, and approved the final version of the editorial.

## References

[B1] Antimicrobial Resistance Collaborators. (2022). Antimicrobial resistance, global burden of bacterial antimicrobial resistance in 2019: a systematic analysis. Lancet. 399, 629–655. 10.1016/S0140-6736(21)02724-0 35065702 PMC8841637

[B2] Sun D. Jeannot K. Xiao Y. Knapp C. W. (2019). Editorial: horizontal gene transfer mediated bacterial antibiotic resistance. Front. Microbiol. 10:1933. 10.3389/fmicb.2019.01933 31507555 PMC6718914

[B3] WHO (2014). Antimicrobial Resistance Global Report on Surveillance. World Health Organization. Available online at: https://www.who.int/publications-detail-redirect/9789241564748

